# Logistic regression models to predict solvent accessible residues using sequence- and homology-based qualitative and quantitative descriptors applied to a domain-complete X-ray structure learning set

**DOI:** 10.1107/S1600576715018531

**Published:** 2015-11-10

**Authors:** Reecha Nepal, Joanna Spencer, Guneet Bhogal, Amulya Nedunuri, Thomas Poelman, Thejas Kamath, Edwin Chung, Katherine Kantardjieff, Andrea Gottlieb, Brooke Lustig

**Affiliations:** a Department of Chemistry, San Jose State University, San Jose, CA 95192-0101, USA; b Department of Mathematics and Statistics, San Jose State University, San Jose, CA 95192-0101, USA; c Department of Biomedical, Chemical and Materials Engineering, San Jose State University, San Jose, CA 95192-0101, USA; d Department of General Engineering, San Jose State University, San Jose, CA 95192-0101, USA; e Department of Chemistry and Biochemistry, Cal Poly San Luis Obispo, San Luis Obispo, CA 93407, USA; f Department of Bioengineering, University of California, San Diego, San Diego, CA 92093-0412, USA; g College of Science and Mathematics, California State University San Marcos, San Marcos, CA 92096-0001, USA

**Keywords:** relative solvent accessibility, logistic regression, Lobanov–Galzitskaya descriptor

## Abstract

A working example of relative solvent accessibility (RSA) prediction for proteins is presented. Novel logistic regression models with various qualitative descriptors that include amino acid type and quantitative descriptors that include 20- and six-term sequence entropy have been built and validated. A domain-complete learning set of over 1300 proteins is used to fit initial models with various sequence homology descriptors as well as query residue qualitative descriptors. Homology descriptors are derived from BLASTp sequence alignments, whereas the RSA values are determined directly from the crystal structure. The logistic regression models are fitted using dichotomous responses indicating buried or accessible solvent, with binary classifications obtained from the RSA values. The fitted models determine binary predictions of residue solvent accessibility with accuracies comparable to other less computationally intensive methods using the standard RSA threshold criteria 20 and 25% as solvent accessible. When an additional non-homology descriptor describing Lobanov–Galzitskaya residue disorder propensity is included, incremental improvements in accuracy are achieved with 25% threshold accuracies of 76.12 and 74.79% for the Manesh-215 and CASP(8+9) test sets, respectively. Moreover, the described software and the accompanying learning and validation sets allow students and researchers to explore the utility of RSA prediction with simple, physically intuitive models in any number of related applications.

## Abstract 2

This article describes the development, validation and application of simple logistic regression models for the prediction of solvent accessible residues in oligomer and non-oligomer sets using a domain-complete X-ray structure learning set.

## 1. Introduction   

The characterization of solvent accessible surfaces is a key task, the results of which are useful in protein design, structural biology and proteomics, notably identifying certain protein–protein interactions. There has been significant effort in the large-scale screening of sequences for characterization of function, including key interactions with ligands and other proteins (Watson *et al.*, 2005 ▸; Tuncbag & Gursoy, 2009 ▸; Zhang *et al.*, 2013 ▸). Possible approaches include the characterization of solvent accessible surfaces directly from sequence information, allowing for the identification of relevant clusters on the surface (Xue *et al.*, 2011 ▸; Tygai *et al.*, 2012 ▸). Other structural features may prove amenable to prediction from sequence, including specifically the identification of critical core hydrophobic residues (Berezofsky & Trifonov, 2001 ▸; Gromiha & Selvaraj, 2004 ▸). Such residues can describe important constraints in modeling protein folding and structure and may be useful when designing modifications for proteins.

Methods using protein sequence information, including first-generation machine learning approaches such as with neural networks, typically have shown a percent accuracy of the order of 70–75% (Rost & Sander, 1994 ▸; Richardson & Barlow, 1999 ▸; Ahmad *et al.*, 2003 ▸). Subsequent methods including large-scale linear regression, support vector regression, support vector machines, *k*-nearest-neighbor analysis and random forest approaches have reported somewhat better results for certain proteins (Wagner *et al.*, 2005 ▸; Wang *et al.*, 2007 ▸; Joo *et al.*, 2012 ▸; Pugalenthi *et al.*, 2012 ▸). The advantage of regression-based approaches is their relative simplicity in both implementation and interpretation. Most recently, a simple, computationally efficient machine learning approach utilizing a general neural network has also been implemented to determine accessible surface area values (Faraggi *et al.*, 2014 ▸).

Linear and generalized linear models can include both quantitative and qualitative predictors, here referred to as ‘descriptors’ (Kutner *et al.*, 2004 ▸). Following our previous work (Rose *et al.*, 2011 ▸; Nepal, 2013 ▸), we include a qualitative descriptor representing query residue type (AA) as well as four homology descriptors: 20-term (E20) and six-term (E6) sequence entropy, fraction of aligned residues that are strongly hydrophobic (FSHP), and fraction of small residue glycine or alanine (FSR). The AA descriptor alone has been shown to be a significant predictor (∼70%) of residue solvent accessibility.

Here we implement logistic regression methods to further elucidate the relationship between simple homology and query sequence descriptors with surface accessibility. We consider both estimation and validation tasks, fitting logistic regression models using learning sets and evaluating our fitted models on test data. Logistic models are one method that should be explored in the context of protein topology, easily incorporating the corresponding quantitative and qualitative descriptors. Logistic regression models have proven to be an adaptable choice for binary classification problems in fields such as economics, epidemiology and the social sciences and are now well established. Logistic approaches have the advantage of directly modeling the probability of success, restricting predicted probabilities to [0, 1] and providing for a natural interpretation in terms of the odds of success.

Our goal is to establish the utility of simple logistic models for the long-established problem of the binary identification of solvent accessible residues. Notably, this is a first step in exploring the functionality of such methods in predicting protein–protein interfaces and interactions from sequence. Such exploration may be accelerated by these methods, given the associated computational complexity.

In addition to providing a working example of such prediction for residue solvent accessibility and applying it to problems associated with protein–protein interaction, we introduce students and researchers in structural biology, biophysical chemistry, bioinformatics and allied fields to an application of logistic regression that includes explicitly both qualitative and quantitative predictors.

Our software (http://www.iucr.org/education/resources) and the accompanying learning and validation sets allow users to explore the utility of residue solvent accessibility (RSA) prediction with simple, physically intuitive models in any number of related applications. Moreover, the corresponding R and Python code can be implemented or otherwise modified to directly predict certain structural features (*e.g.* possible protein–protein interfaces), and new test sequences can be explored by simple implementation of NCBI BLASTp (http://blast.ncbi.nlm.nih.gov/Blast.cgi?PAGE=Proteins) and the Protein Data Bank (http://www.rcsb.org/pdb/home/home.do).

## 2. Methods   

### 2.1. Sequence entropy and other homology-based descriptors   

As an alternative to the standard 20-term (E20) sequence entropy (Gerstein & Altman, 1995 ▸) we calculated the six-term (E6) sequence entropy (Mirny & Shakhnovich, 1999 ▸). The entropy terms are summed over six classes of amino acids (*e.g.* polar), where the sequence entropy at the *k*th residue position is expressed as 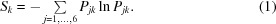
Here the probability *P_jk_* at amino acid sequence position *k* is derived from the frequency for an amino acid class *j* at sequence position *k* for *N* aligned residues. Including gaps as the 21st element in our original 20-term sequence entropy calculations was problematic (Liao *et al.*, 2005 ▸). Thus, we treated the remaining sequence homology terms, fraction strongly hydrophobic (FSHP) and small residue (FSR), as just fractions of aligned residues. Note the strongly hydrophobic residues (*i.e.* V, L, I, F, Y, M, W), and the remaining 13 are the non-strongly hydrophobic residues.

### 2.2. Data assembly   

**Figure 1:**
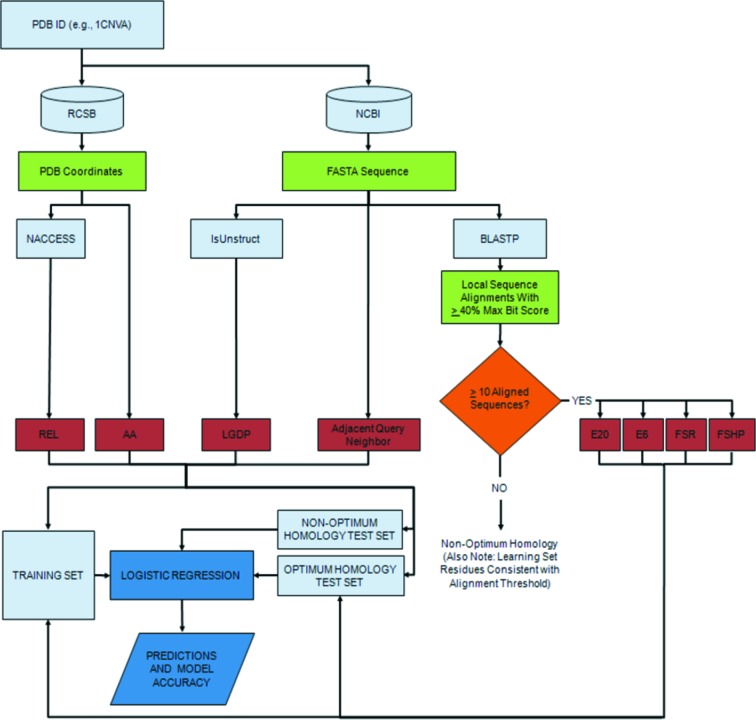
Flowchart of key inputs and outputs.

Our original learning set of 268 protein chains included query proteins with known three-dimensional X-ray crystallographic structure and was confirmed to be a diverse, representative set (Mishra, 2010 ▸). A more extensive learning set of 1363 protein chains (Nepal, 2013 ▸) was similarly selected with a resolution < 2.5 Å and an *R* value < 0.3 using *PISCES* (Wang & Dunbrack, 2003 ▸) from an exhaustive list involving some 6500 protein domains (Bondugula *et al.*, 2011 ▸). Redundant and other problematic sequences were also culled for chains with percent identities greater than 25. To determine our sets of aligned protein sequences and their individual residue elements, we chose a straightforward and non-biased standard application of BLASTp (Altschul *et al.*, 1997 ▸) to a non-redundant database (GenBank; http://www.ncbi.nlm.nih.gov/genbank/), with *BLOSUM62* (Henikoff & Henikoff, 1992 ▸) allowing for bit scores greater than or equal to 40% of the best score (see Fig. 1 ▸). Test sets are similarly treated. Corresponding homology-based calculations such as E20 using *psi-BLAST* (Altschul *et al.*, 1997 ▸) showed little added value in prediction accuracy. Alternative applications of multiple sequence alignment using *MUSCLE* (Edgar, 2004 ▸) indicated no significant differences in accuracy (E. Chung & B. Lustig, unpublished results).

We continue with simple approaches to parsing homology data, noting that requiring at least ten aligning BLASTp subject sequences given the 40% threshold relative to the highest bit score is a reasonable condition for fully reliable sequence entropies (Liao *et al.*, 2005 ▸; Rose *et al.*, 2011 ▸). We validate our models using two test sets, where one standard test set, Manesh-215 (Naderi-Manesh *et al.*, 2001 ▸), has been thoroughly evaluated with respect to many standard RSA approaches (Nguyen & Rajapske, 2006 ▸). Additionally, we evaluated the models on the more recent CASP(8+9), a comparably sized test set evaluated by more current methods (Joo *et al.*, 2012 ▸). However, for the Manesh-215 and CASP(8+9) test sets we are required to use a non-optimum homology subset, given that the corresponding sequence and coordination data are listed externally. Fourteen such proteins are noted for the Manesh-215 set, six proteins from the set of 102 X-ray structures of CASP8 and 12 proteins from the set of 88 X-ray structures of CASP9 (see Table 1 ▸).

### 2.3. Nearest neighbors and propensity for disorder   

One simple and physically intuitive approach to account for the effects of sequence neighbors is to utilize the Ising approach of Lobanov *et al.* (2012 ▸). Here the propensity for each residue being disordered, the Lobanov–Galzitskaya disorder probability (LGDP), is introduced as an additional quantitative descriptor (*IsUnstruct*; http://bioinfo.protres.ru/IsUnstruct/). Calculations of Lobanov–Galzitskaya propensities include a very limited number (<2%) of FASTA (http://www.ncbi.nlm.nih.gov/BLAST/blastcgihelp.shtml) neighbors not included in the PDB coordinates. Undefined FASTA residues, typically less than 1% of our sequences, are assumed to be alanine for the purposes of adjacent neighbor analysis only. Also, specifically the two sequence adjacent residues, adjacent query neighbors (AQN), are defined by FASTA and can be treated as qualitative descriptors. All regression documented here is scalable on a single E5410 Intel Xeon 4P Linux box or comparable 64-bit laptop. Lobanov–Galzitskaya Ising-model calculations appear algorithmically straightforward and are not significantly intensive.

### 2.4. Surface accessibility   

One gold standard for validation of solvent accessibility predictions is the RSA values determined from the query X-ray structures using *NACCESS* (Lee & Richards, 1971 ▸; Hubbard & Thornton, 1993 ▸). Alternative calculations using DSSP-based (Kabsch & Sander, 1983 ▸; Ahmad *et al.*, 2004 ▸) solvent accessibility data showed no significant differences in prediction accuracy (J. Spencer, B. Lustig & A. Gottlieb, unpublished results). Here we initially treated amino acids with less than 20% relative exposure to solvent (RSA) as buried (Carugo, 2000 ▸). An alternative threshold of 25% was subsequently explored. Model accuracies are estimated on the basis of the standard expression of Richardson & Barlow (1999 ▸), where the accuracy is 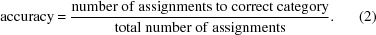
Solvent accessibility for interdomain (interface) regions between chains is directly evaluated from the relevant PDB (RCSB or PISA-PDBe) coordinate files *via* *NACCESS*, consistent with Bahadur *et al.* (2004 ▸). The 1363-protein learning set is considered diverse, robust and exhaustive (Nepal, 2013 ▸). Very conservative application of the biological unit as prescribed by PISA-PDBe (http://www.ebi.ac.uk/pdbe/pisa/) has been implemented in the test sets to optimize the reliability of their evaluation for accuracy. Only unambiguously oligomeric protein structures [88 for Manesh-215, 123 out of 200 for CASP(8+9) from PISA-PDBe, with a biological unit different from the asymmetric unit, 66 and 80 for Manesh-215 and CASP(8+9), respectively] are noted as such for analysis.

### 2.5. Logistic regression   

Here we directly model the probability of accessibility utilizing the naturally dichotomous outcome variable, accessibility status. This approach avoids the common pitfalls of inappropriately applying multiple regression to percentage data, such as obtaining negative probabilities or probabilities that exceed 1. We assume the independence of all residues, although in reality there is some correlation between neighbors. A logistic regression model using residue solvent accessibility as a dichotomous response with *Y* = 0 for buried residues and *Y* = 1 for accessible residues was implemented.

Logistic models are a generalization of multiple linear regression in which a binary dependent variable is described as a function of both quantitative and qualitative variables (Kutner *et al.*, 2004 ▸; Hosmer *et al.*, 2013 ▸). We utilized four quantitative descriptors and 19 dummy variables corresponding to the descriptor AA, such that linear predictor is of the form 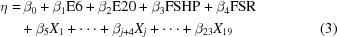
for *j* = 1,…, 19. Here *X_j_* are the indicator variables associated with qualitative descriptor AA. We used treatment coding with Val as the baseline treatment *via* R 3.1.0, where the *X_j_* values are either 0 or 1, with 1 corresponding to the given amino acid type.

**Figure 2:**
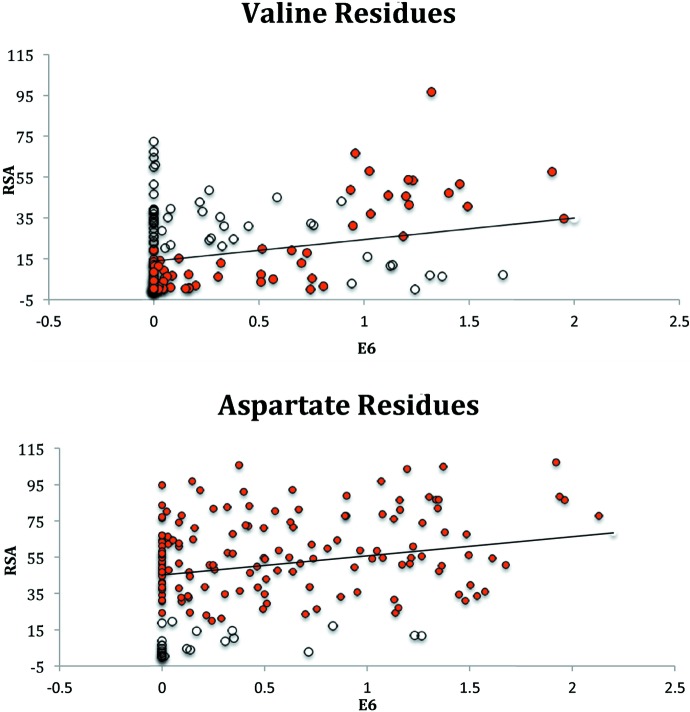
Linear and logistic regression fits for query residues valine (V) and aspartate (D) from the 18-protein transient-binding subset. Here, the least-squares fit corresponds to the *NACCESS* RSA values regressed on E6 and amino acid type (AA). For illustrative purposes only two amino acid types are shown. Valine (top) and aspartate (bottom) include 177 and 172 residues, respectively. Both least-squares fits have a slope (E6) of 10.56, but they have different corresponding intercepts 13.83 and 45.17. The residues correctly classified by the logistic model (E6+AA) are shown in red (127 for V, 148 for D). Note, 76.49 (linear) and 75.64% (logistic) of all 2786 residues are classified correctly. Here, a 20% threshold was utilized in both observed and predicted RSA values to create classifications. Moreover, the results were validated by evaluating the fitted model on a 13-protein subset (2049 residues) of the Manesh-215 test set consisting of transient-binding proteins (Pettit *et al.*, 2007 ▸). Here we observe slightly higher accuracies of 76.34 (linear) and 77.27% (logistic).

Let *p* denote the probability that a particular residue is accessible and 1 − *p* the probability of a residue being buried. Under the logistic regression framework, the odds of success, *p*/(1 − *p*), are modeled as an exponentiated linear combination of the descriptors: 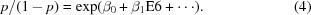
The probability that a particular residue is accessible can be expressed by rearranging the terms in equation (4), so that 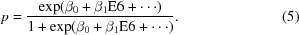
Estimates for the model parameters are obtained using maximum likelihood methods, determining parameters that optimize the likelihood of the observed data (consisting of zeros and ones) being described by the model (Long, 1997 ▸). The probability distribution function associated with equation (5) is an S-shaped curve and a more realistic representation of probabilities associated with binary outcomes. The corresponding fitted values are probabilities, which then can be converted to classifications of either 1 or 0 using a 0.5 standard threshold. Shown in Fig. 2 ▸ are the logistic regression predictions superimposed on the least-squares regression fit for the 18-protein transient-binding set as identified by Pettit *et al.* (2007 ▸).

The expression in equation (5) is commonly referred to as the odds, where the difference between two log odds can be expressed as 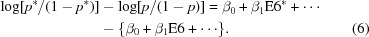
As an example, we assume all other non-E6 terms are held constant such that the ratio of the odds of success corresponding to a increase of 1 entropy unit, so that E6* = E6 + 1, is given by 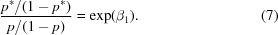
Table 2 ▸ reports the results of a logistic regression fit using the 1363-based learning set. Included are the parameter estimates, corresponding standard errors, and *z* and *p* values based on the Wald test (Ghosh & Sen, 1991 ▸). We find that all of the continuous descriptors and nearly all of the dummy variables associated with the categorical descriptor are significant at a 0.05 significance level.

### 2.6. Learning and test sets   

As a first example, included for pedagogical purposes, we fit a logistic model with a qualitative descriptor describing all 20 amino acid types (AA) and a quantitative predictor E6 with the results shown in Fig. 2 ▸. The learning set corresponds to all 18 available transient-binding proteins from our original 268 learning set (Mishra, 2010 ▸; Rose *et al.*, 2011 ▸) and current 1363-based learning set. These transient-binding proteins were originally characterized as such by Pettit *et al.* (2007 ▸). Included in this figure is the least-squares fit with slope of 10.56 for each amino acid type and varying intercepts (Val and Asp samples are shown in Fig. 2 ▸). The logistic fit generates probabilities, which allows for binary classification of all residues as buried *versus* solvent accessible. We then analysed the accuracies for 13 transient-binding proteins similarly identified from the standard Manesh-215 test set.

Our next step is to build various models based on the domain-complete 1363-protein learning set and validate them on the complete standard Manesh-215 test set and the more recent CASP(8+9). We were initially interested in evaluating our models on relevant subsets of the test sets, in particular, transient-binding proteins. However, defining transient-binding proteins is not currently a consensus exercise (Acuner-Ozbabacan *et al.*, 2011 ▸). Here, we make a more conventional delineation between oligomers and non-oligomers that is effectively universal in its application. A more complete examination of oligomers and the remaining non-oligomers is warranted.

### 2.7. Specific strategies for optimum homology *versus* non-optimum homology proteins   

As shown in the flowchart (see Fig. 1 ▸) there is a key branch point involving test sets being partitioned into an optimum homology (*i.e.* ten or more aligned sequences) subset and a remaining non-optimum homology subset (see Table 1 ▸ for complete list). The reason is that homology-based descriptors are not reliable predictors for non-optimum homology proteins, where we only validate those models with exclusively non-homology descriptors. The resulting strategy is to use models with exclusively non-homology descriptors for the non-optimum proteins. Therefore, we report a weighted average for all proteins based on the accuracies of two components: (1) predictions for optimum homology proteins from models that include both homology-based descriptors (*e.g.* E6) and non-homology ones (*e.g.* LGDP); (2) predictions for non-optimum homology proteins from models including exclusively non-homology descriptors.

## 3. Results and discussion   

### 3.1. General   

We have evaluated various models with different subsets of the descriptors. Table 2 ▸ reports an initial regression for the saturated E20+E6+FSR+FSHP+AA model (referred to here as the classic model). Note that for this logistic regression E20 and E6 were shown in the corresponding correlation matrix output to be 83% correlated. Models showing at least 70% accuracy indicate comparable standard errors and *p* values based on the Wald test to the saturated model illustrated in Table 2 ▸. However, it is clear that descriptors FSR and FSHP are somewhat unique in their pattern of being primarily 1 or 0 in value (Mishra, 2010 ▸). So, most recently Box–Tidwell power transformations were applied to test/optimize log-linearity. The resulting relative order and sign of the β regression coefficients, compared to those originally determined for Table 2 ▸, remain unchanged and no significant increases in resulting classification accuracy are indicated.

Table 3 ▸ reports the accuracies for a family of models for the optimum homology subsets for the Manesh-215 and CASP(8+9) test sets, and Table 4 ▸ reports the accuracies of models also evaluated on oligomer and non-oligomer subsets. The classic model of Table 2 ▸ remains the best homology-based model in terms of prediction accuracy. However, we note the high correlation (0.83) between E6 and E20, unlike other quantitative predictors. Also, even the simpler E6+AA model performs nearly as well for both the 20% and the alternative 25% RSA threshold. In both the multiple linear regression (Rose *et al.*, 2011 ▸; Nepal, 2013 ▸) and logistic models, E6 appears to be the more reliable predictor of residue solvent accessibility as opposed to E20 (see Table 3 ▸). This is consistent with the notion that E6 entropy is the more sensitive measure of residue packing (Mirny & Shakhnovich, 2001 ▸).

We augmented our set of descriptors with several query sequence-based variables. Inclusion of the LGDP quantitative descriptor and two AQN qualitative descriptors describing the amino acid type for the two sequence-adjacent amino acids generated incremental associated gains in accuracy (Table 4 ▸). A comprehensive model consisting of sequence homology-based descriptors (E6, FSR, FSHP) and sequence-based descriptors (AA, LGDP, AQN) was found to produce the highest accuracies for both Manesh-215 and CASP(8+9) optimum homology sub-sets. Note that the total all-proteins accuracies are, for the respective 25 and 20% thresholds, 76.11 and 76.00 for Manesh-215, and 74.79 and 74.55% for CASP(8+9).

Moreover, non-oligomers are often associated with higher accuracy than the oligomers (see Table 4 ▸). This is especially true when looking at models with a significant homology-based descriptor component. More importantly, the removal of likely interfacial residues resulted in slightly better accuracies. Here such a set comprising about one-fifth of the total oligomer residues was determined from residues showing measurable increases in single-chain *NACCESS* values when compared to the corresponding oligomer ones. As a typical example the likely interfacial residues for the all-proteins model show a reduced accuracy with a 25% threshold of 71.66 and 70.35 for Manesh-215 and CASP(8+9), respectively.

### 3.2. Implementation of simple logistic models   

In this study, the initial focus has been the examination of sequence- and homology-based descriptors in logistic regression models for protein residue accessibility. Here, a logistic model including only the E6 descriptor sequence entropy for a particular sequence position in conjunction with AA, the amino acid type, offers nearly the same degree of prediction accuracy as the classic model that also includes E20 entropy and the fraction of aligned residues that are strongly hydrophobic (FSHP) as well as being small (FSR). But E6 and E20 are highly correlated, so we preferred the former descriptor because of its generally better prediction capabilities. We have demonstrated that the inclusion of an additional quantitative descriptor, the LGDP, gives associated gains in prediction accuracy of the order of 1%, while the adjacent neighbor descriptors give somewhat less associated gain in prediction accuracy. Certainly, further model building including other simple descriptors is a reasonable next step. One could even consider further refinement of the coordinate data sets themselves based on established structure validation criteria (Reddy *et al.*, 2003 ▸; Read *et al.*, 2011 ▸). Though computationally intensive, it is a one-time event and does not interfere with the model simplicity.

Even the most computationally intensive machine-learning methods involving optimal threshold RSA criteria show binary prediction limits at or below 80% in accuracy (Joo *et al.*, 2012 ▸). Indeed, our logistic regression models with homology-based and qualitative query sequence descriptors compare favorably with existing single-stage methods and other approaches that preclude an extensive machine-learning task (Gianese *et al.*, 2003 ▸). Simple models have great utility, including computational simplicity and efficiency. Prioritization of parsimony in model selection is consistent with the Pareto principle, suggesting that any model for which some 80% of outcomes can be described by 20% of the causes is preferable (Pareto, 1971 ▸). This prioritization may be required for the greater challenges associated with very large scale calculations involving the applications of these and related models to exhaustive searches of the proteome, including the search for possible protein–protein interactions.

### 3.3. Intrinsic limitations and future opportunities   

#### 3.3.1. General   

There appears to be a fundamental limitation for prediction accuracy for surface accessible residues, which probably involves the coupled nature of surface residue accessibility with inter-chain interactions. This constraint is similar to the intrinsic limitation of secondary structure prediction (Rost, 2001 ▸), which involves the coupling of secondary with tertiary structure (Kihara, 2005 ▸). Specifically, chain residue accessibility prediction is complicated by hydrophobic residues being found not only in core positions but also on surfaces involving interactions with other protein chains (Yan *et al.*, 2008 ▸). Prediction of solvent accessibility is further obfuscated by the still largely uncharacterized nature of transient and related protein interactions. It is clear, however, that at least for oligomers, when likely interface residues are removed, small but measurable increases in accuracy are indicated. This unique character for such residues is consistent with previously detected residue conservation signals for oligomeric interface regions (Elcock & McCammon, 2001 ▸; Valdar & Thornton, 2001 ▸; Guharoy & Chakrabarti, 2005 ▸). Interestingly, the 1363-based learning set partitioned into oligomers and non-oligomers showed significantly less accuracy for their respective application to the oligomers and non-oligomers.

Some 5% of PDB residues are identified as missing and possibly disordered and are a non-trivial component of the actual protein chains (Brandt *et al.*, 2008 ▸; Lobanov *et al.*, 2010 ▸). These generally correspond to residues not identified in an X-ray structure. We note, at least for the aligned portions of FASTA and PDB sequences, a comparable number of such unaccounted residues exist. Of course, no gold standard such as the simple application of *NACCESS* is available for these types of residues. However, in the future as we continue to build models predicting protein–protein interfaces, the inclusion of such disordered residues is of likely advantage (Mészáros *et al.*, 2009 ▸). Our successful incorporation of an intrinsic residue disorder propensity as a descriptor suggests the suitability of this sort of approach in related problems, notably the prediction of protein interfaces with respect to other proteins, nucleic acids and small ligands.

Large-scale exploration of sequence space, generated off a set of structural ensembles for three-dimensional protein structures, has shown clustering of sequence entropy values corresponding to a particular fold (Larson *et al.*, 2002 ▸). In a similar fashion, Shannon entropies for protein sequence have been shown to correlate with configurational entropies calculated from local physical parameters, including backbone geometry (Koehl & Levitt, 2002 ▸). Our earlier calculations indicated a correlation between sequence entropy and inverse packing density (a measure of local flexibility) as well as query hydrophobicity (Liao *et al.*, 2005 ▸; Mishra, 2010 ▸). Here we can reasonably assume that there are a cohort of very buried residues that are both well packed and not likely to have any portion of their surface area accessible to solvent.

#### 3.3.2. Exploring additional features of protein structure in the classroom with logistic regression   

For MAML-1 and other proteins, the propensity of residue disorder as a possible indicator of local environments was explored as part of a graduate course seed project (Nedunuri, 2013 ▸). A broader exploration of proteins involving the 18 learning set and 13 test set transient-binding proteins (noted in Fig. 2 ▸) was made in the next such course offering, integrating the related LGDP descriptor into prediction and further classification of solvent accessible residues (Kadiyala, 2015 ▸). Logistic regression outputs such as those indicated by Table 2 ▸ can be considered tools for exploration. Note, for instance, the very large and negative β regression coefficient of −1.543 for Cys, which is unique among the AA qualitative descriptors. Such a value is consistent with Cys forming a tertiary contact (Dosztányi *et al.*, 1997 ▸) and the fact that residues most likely to form tertiary contacts are significantly less likely to have side chains accessible to solvent (Kim & Park, 2004 ▸).

## 4. Conclusion   

Homology- and sequence-based descriptors can be used to build classifiers of residue accessibility. We establish their utility as both quantitative and qualitative descriptors in logistic regression models, demonstrating competitive accuracy using as few as two descriptors. Removal of likely oligomeric interfacial residues typically increases prediction accuracies, suggesting specific homology-based and other signals for such interfaces. In predicting solvent accessible residues, E6 sequence entropy is a very useful descriptor, consistent with the notion that to a first approximation highly conserved residues are typically found in the interior of the protein. Moreover, such conserved residues probably involve hydrophobic residues given their tendency to be buried. But also some conserved residues can be partitioned on the surface, accessible to solvent, and this includes some that are hydrophobic. Our methods are well suited for simple integration of homology variables with a variety of potential sequence and other sequence neighbor descriptors, allowing for application of a variety of models, including new ones, to even more challenging computational problems involving the characterization of protein interfaces and protein–protein interactions.

## Figures and Tables

**Table 1:** Non-optimum homology subsets for test set proteins

Manesh-215	CASP8	CASP9
1axna	3d3oa	3mqza
1bhmb	3d5pa	3n53a
1ceoa	3dewa	3n6za
1cnva	3df8a	3na2a
1esca	3dm3a	3ngwa
1exnb	3doua	3ni8a
1hlba		3njaa
1kpta		3nkga
1udii		3nrga
1vcaa		3nrva
1wbaa		3nwza
2ccya		3nyma
2scpa		
2sila		

**Table 2:** Classic model fit (E20+E6+FSR+FSHP+AA) including parameter estimates, corresponding standard errors, and *z* and *p* values based on the Wald test Descriptors included are sequence entropies E20 and E6, the amino acid qualitative predictor (AA) with 20 classes, and FSHP and FSR indicating the fraction of optimum homology residues that are strongly hydrophobic (V, I, L, F, M, Y, W) and small (A, G). The standard 1363-based learning set and a 20% threshold was utilized.†

Variables	β	Standard error	exp(β)	*z* value	*P* (>|*z*|)
Intercept	−0.528	0.031	0.590	−17.247	<0.001
E20	0.342	0.012	1.407	29.162	<0.001
E6	0.862	0.017	2.369	51.156	<0.001
FSR	−0.922	0.031	0.398	−29.690	<0.001
FSHP	−1.646	0.030	0.193	−54.898	<0.001
ALA	−0.267	0.034	0.766	−7.861	<0.001
ARG	0.765	0.034	2.149	22.363	<0.001
ASN	0.358	0.035	1.430	10.236	<0.001
ASP	0.774	0.034	2.168	22.670	<0.001
CYS	−1.543	0.052	0.214	−29.405	<0.001
GLN	0.366	0.036	1.442	10.234	<0.001
GLU	0.985	0.034	2.677	29.047	<0.001
GLY	0.829	0.038	2.292	21.588	<0.001
HIS	−0.114	0.038	0.893	−3.003	0.003
ILE	−0.036	0.027	0.965	−1.353	0.176
LEU	0.202	0.023	1.224	8.767	<0.001
LYS	1.509	0.036	4.522	41.384	<0.001
MET	0.269	0.036	1.308	7.424	<0.001
PHE	0.040	0.030	1.041	1.359	0.174
PRO	0.449	0.034	1.567	13.072	<0.001
SER	−0.166	0.032	0.847	−5.113	<0.001
THR	−0.168	0.032	0.845	−5.291	<0.001
TRP	0.567	0.041	1.763	13.784	<0.001
TYR	0.690	0.029	1.995	24.195	<0.001

† Note descriptor values for nine PDB chains (1G291, 1L2WA, 1MUWA, 1W85I, 1XC3B, 1XVHA, 2I6CA, 2PI2E) from the original 1363 set are insufficient and here considered null.

**Table 3:** Selected logistic model accuracies for test sets based on X-ray crystal structures For comparison, accuracies are shown for models built using both 20 and 25% relative solvent accessibility threshold values. The standard 1363-based learning set was utilized for model fitting.

	1363 training/Manesh-215 test	1363 training/CASP(8+9) test
Model	Optimum homology†	Optimum homology‡
Threshold	25% *20%*	25% *20%*
E20	66.10 *64.74*	64.81 *63.50*
E6	69.40 *69.18*	68.06 *67.50*
FSHP	65.61 *67.42*	66.14 *68.23*
AA	69.62 *70.36* (69.48 *71.57*)§	68.36 *70.11* (68.89 *71.86*)¶
E6+AA††	74.79 *74.78* (69.35 *69.68*)	73.51 *73.76* (*63.98 67.23*)
Classic‡‡	75.56 *75.09* (69.*64 69.90*)	74.32 *74.23* (65.47 *67.66*)

† Optimum homology Manesh-215 subset (47 609 residues).

‡ Optimum homology CASP(8+9) subset (41 967 residues).

§ Non-optimum homology Manesh-215 subset (3113 residues).

¶ Non-optimum homology CASP(8+9) subset (2832 residues).

†† Note the other two models with AA and a single quantitative descriptor, E20+AA and FSHP+AA, are not reported as they have less predictive accuracy than E6+AA.

‡‡ As shown in Table 2 ▸, E20+E6+FSR+FSHP+AA saturated model.

**Table 4:** Selected logistic model accuracies for test sets based on X-ray crystal structures LGDP and AQN are included as additional descriptors. We list prediction accuracies for oligomers and non-oligomers together. The standard 1363-based learning set was utilized for model fitting. The difference in accuracy for oligomers minus non-oligomers is scaled, M if difference <−0.5, P if >0.5 and otherwise O. The change in total accuracy for oligomers with likely interfacial residues removed is scaled M if difference <−0.5, P if >0.5 and otherwise O.

	1363 Training/Manesh-215 test		1363 Training/ CASP(8+9) test	
	Optimum homology†		Optimum homology‡	
Model	Total Acc 25% *20*%	ΔAcc (Olig-NonOlig) // ΔAcc (Olig w/o interface) 25% *20*%	Total Acc 25% *20*%	ΔAcc (Olig-NonOlig) // ΔAcc (Olig w/o interface) 25% *20*%
E6+FSR+FSHP+AA	75.23 *74.95*	M *M* // P *P*	74.12 *74.15*	M *M* // P *P*
E6+AA	74.79 *74.77*	O *M* // P *P*	73.50 *73.77*	O *M* // P *P*
LGDP	60.03 *58.51*	P *P* // M *M*	56.99 *56.10*	P *P* // M *O*
AQN(*i* − 1)	55.97 *52.25*	P *P* // M *M*	52.45 *49.98*	P *O* // M *M*
AQN(*i* + 1)	55.46 *52.72*	P *P* // M *M*	51.89 *51.34*	P *O* // M *M*
LGDP+E6+AA	75.74 *75.57*	O *M* // P *P*	73.92 *74.05*	O *M* // P *P*
LGDP+E6+FSR+FSHP+AA	76.05 *75.90*	O *M* // P *P*	74.56 *74.53*	M *M* // P *P*
LGDP+AA+AQN[Table-fn tfn10] [Table-fn tfn11]	71.29 *71.36*	P *O* // P *P*	69.75 *69.99*	P *O* // P *P*
Comprehensive model[Table-fn tfn12]	76.41 *76.28*	M *M* // P *P*	75.01 *74.76*	O *M* // P *P*
All proteins[Table-fn tfn13]	76.11 *76.00*	O *M* // P *P*	74.79 *74.55*	M *M* // P *P*

† Optimum homology Manesh-215 subset for oligomers (21 513 residues; 16 283 residues non-interfacial) and non-oligomers (26 096 residues); alignment with LGDP values truncated 132 of 146 residues for 8ATCB, and one residue each for 1CHMA and 1TYSA.

‡ Optimum homology CASP(8+9) subset for oligomers (24 176 residues; 18 573 residues non-interfacial) and non-oligomers (17 791 residues).

§ Non-homology descriptor model evaluated on non-optimum homology Manesh-215, gives percent accuracies of 73.12 (25% threshold) and *71.49* (*20*% threshold), for oligomers (919 residues; 787 residues non-interfacial); 70.95 (25% threshold) and *71.75* (*20*% threshold) for non-oligomers (2194 residues).

¶ Non-homology descriptor model that, when evaluated on non-optimum homology CASP(8+9), gives percent accuracies of 72.26 (25% threshold) and *71.83* (*20*% threshold) for oligomers (2080 residues; 1393 residues non-interfacial); 69.55 (25% threshold) and *70.88* (*20*% threshold) for non-oligomers (752 residues).

†† E6+FSR+FSHP+AA+LGDP+AQN model.

‡‡ Residue weighted accuracies, comprehensive model for optimum homology proteins and non-homology descriptors for non-optimum homology proteins.
